# Life Cycle Exposure to Cyhalofop-Butyl Induced Reproductive Toxicity in Zebrafish

**DOI:** 10.3390/toxics10090495

**Published:** 2022-08-25

**Authors:** Manman Duan, Xuanjun Guo, Xiangguang Chen, Mengyu Guo, Hao Xu, Lubo Hao, Chengju Wang, Yang Yang

**Affiliations:** 1 Innovation Center of Pesticide Research, Department of Applied Chemistry, College of Sciences, China Agricultural University, Beijing 100193, China; 2 State Key Laboratory for Biology of Plant Disease and Insect Pests, Institute of Plant Protection, Chinese Academy of Agricultural Sciences, Beijing 100193, China

**Keywords:** cyhalofop-butyl, reproduction, HPGL axis, environmental concentrations

## Abstract

Cyhalofop-butyl (CyB) is a herbicide widely used in paddy fields that may transfer to aquatic ecosystems and cause harm to aquatic organisms. In this study, zebrafish (Danio rerio) were exposed to CyB at environmental concentrations (0.1, 1 and 10 µg/L) throughout their adult life cycle, from embryo to sexual maturity. The effects of CyB on zebrafish growth and reproduction were studied. It was found that female spawning was inhibited, and adult male fertility decreased. In addition, we examined the expression of sex steroid hormones and genes related to the hypothalamus–pituitary–gonad–liver (HPGL) axis. After 150 days of exposure, the hormone balance in zebrafish was disturbed, and the concentrations of 17β-estradiol (E2) and vitellogenin (VTG) were decreased. Changes in sex hormone were regulated by the expression of genes related to the HPGL axis. These results confirmed that long-term exposure to CyB at environmental concentrations can damage the reproductive capacity of zebrafish by disrupting the transcription of genes related to the HPGL axis. Overall, these data may provide a new understanding of the reproductive toxicity of long-term exposure to CyB in zebrafish parents and offspring.

## 1. Introduction

Cyhalofop-butyl (CyB) is an aryloxyphenoxypropionate (APP) herbicide widely used in rice paddies around the world, first manufactured by Dow AgroSciences in 1987 [1,2]. It was first introduced in Asia in 1995 [3,4] and has been widely used in this type of agriculture for more than twenty years [5]. In China, it has been registered as an herbicide for selectively controlling weeds in rice fields in 2006 and is increasingly used in agricultural production [6,7,8], which has led to its easy transfer to the aquatic environment. Therefore, the potential hazards due to the spread of CyB in aquatic organisms should be investigated. The detected concentration of CyB in bulk drainage water samples in Japan is 0.01–0.08 μg/L [2,9]. The situation is even worse in China. In fact, in southern China, after spraying 135 g a.i./ha of 10% fenoxaprop-p-ethyl•cyhalofop-butyl EC herbicide, the detected concentration of CyB can be as high as 2.017 mg/L in rice fields [10]. Some studies have reported the effects of CyB on aquatic organisms. For example, CyB can induce developmental toxicity, oxidative stress and apoptosis in zebrafish embryos [2,11,12] as well as other aquatic organisms, such as *Yellow River Carp* [13], *Rana chensinensis tadpole* [14] and *Misgurnus anguillicaudatus* [7]. Knowledge of the reproductive toxicity of CyB in aquatic organisms, especially fish, and its potential mechanism is very limited.

The measurement of steroid hormones in teleost is very useful for monitoring the reproductive system, because changes in these hormones play a vital role in fish reproduction and correspond to changes in the expression of genes involved in the steroid production pathway [15,16]. Moreover, reproductive capacity and individual development are coordinated by the interaction of genes associated with the hypothalamus–pituitary–gonad–liver (HPGL) axis [17,18,19,20]. Changes in sex steroid concentrations may cause reproductive dysfunction such as sex differentiation, fertility and fertilization rate declines [21]. Gonadotropins secreted by the pituitary gland (such as follicle-stimulating hormone, FSH, and luteinizing hormone, LH) act on the gonads to regulate sex hormone synthesis and gametogenesis. In organisms, FSH and LH regulate the synthesis of 17β-estradiol (E2) and testosterone (T), which participate in gametogenesis and oviposition [22,23]. In zebrafish gonads, *cyp17* converts 17-hydroxyprogesterone into androstenedione, which is then converted to T in a reaction catalyzed by *17βhsd* [24]. T, secreted by the follicles, is converted into estradiol by aromatase (cyp19a) [25,26]. Therefore, E2 and T disorders will affect fish reproduction. E2 enters the liver through the blood circulation system, stimulating and promoting the synthesis and secretion of vitellogenin (VTG) [27]. VTG travels through the bloodstream to the ovary, where it is decomposed into yolk proteins that provide sufficient nutrients for the growth and development of offspring. The synthesis and secretion of these steroid hormones promote sex differentiation, the growth and development of the gonads, and finally regulate their reproductive system [28,29].

Our research aimed to assess the reproductive toxicity of CyB in zebrafish by exposing zebrafish embryos (2 h post fertilization), in comparison to a control group, to nominal concentrations (0. 1, 1 and 10 μg/L) of CyB for 120 days until adulthood and analyzing the potential impact of CyB on the whole life cycle of zebrafish. The reproductive capacity, sex gland index (GSI), sex steroid hormones and plasma VTG concentrations, gonad histology and the relative mRNA levels of genes related to the HPGL axis were measured. In addition, differences in female and male zebrafish responses to CyB are discussed. The results of this study will contribute to a better understanding of the negative effects of CyB on fish reproduction and their underlying mechanisms.

## 2. Materials and Methods

### 2.1. Chemicals

Cyhalofop-butyl (CAS: 122008-85-9) (97.5%) was obtained from Jiangsu Zhongqi Technology Co., Ltd. (Jiangsu, China) and dissolved in dimethyl sulfoxide (DMSO) to obtain a stock solution. All other reagents were of analytical grade.

### 2.2. Zebrafish Rearing and CyB Exposure

The parental zebrafish of the wild-type AB strain were purchased from the Beijing Hongda Gaofeng Aquarium Department, China. After domestication for two weeks, the parents were cultured in a flow-through feeding facility (Esen Corp. Beijing, China) at a temperature of 27 ± 1 °C and a photoperiod of 14:10 h (light/dark) and were fed with live brine shrimps three times a day. Embryo exposure experiments were performed according to OECD guidelines and methods described in previous studies [30,31], ensuring that the final DMSO volume was less than 0.01% (*v*/*v*). Two hours after fertilization, 1200 normal embryos obtained by mating in the spawning box were randomly divided into 4 treatment groups (control group, 0.1, 1 and 10 μg/L) and transferred to 2.5 L containers (3 repetitions of each treatment). Each container contained 100 embryos and 1 L of exposure solution. Reconstituted water (1.27 mM NaHCO_3_, 0.33 mM MgSO_4_, 0.33 mM CaCl_2_ and 0.17 mM KCl) with pH 7.5 ± 0.5 was used to make the exposure solution, which was changed every day. The exposure method was based on research in the literature [32]. At 30 days after fertilization (dpf), zebrafish were transferred to a 5 L water tank. At 60 dpf, about 1200 zebrafish were transferred to 20 L containers to obtain four treatment groups (control group, 0.1, 1 and 10 μg/L). Each container contained about 50 fish and 20 L of exposure solution (each treatment was performed 3 times, and each repetition included two containers). The fish were subjected to continuous exposure for 120 d. During exposure, zebrafish were fed according to the method of Mu, Qi [33] as described in the Supplementary Materials (Appendix A). After 120 days of exposure, 16 pairs of zebrafish in each treatment group were randomly selected for a reproduction test, and four replicates were set at the same time. Eggs were laid weekly, and embryos were collected and counted 1 hour after fertilization. All animal experiments were conducted under the policy of the Animal Ethics Committee of China Agricultural University.

At the end of the spawning experiment (150 d), the spawning fish were subjected to fasting for 24 h and then euthanized with 0.03% MS-222. After collecting the blood of female and male zebrafish and recording body weight, body length, and the weights of the brain, liver, and gonad tissues, the samples were frozen at −80 °C and then utilized for a gonad histopathological examination and RNA expression analysis (3 replicates/group, 5 fish/replicate), as well as for the determination of the brain body index (BSI), liver body index (HSI) and gonad index (HSI) (3 replicates/group, 50 fish/replicate). The collected blood samples were used to determine the levels of sex steroid hormones (E2 and T) and vitellogenin (VTG).

### 2.3. Gonadal Histological Examination

Histological specimens of zebrafish gonads were prepared according to previous methods [34], and histological examination was conducted using an Olympus microscope (Olympus, Japan).

### 2.4. E2, T, 11-KT and VTG Concentrations Measurement

Enzyme-linked immunosorbent assay (ELISA) kits (Yanjin Biological Co., Ltd., Shanghai, China) were used. Briefly, blood samples were centrifuged (4 °C, 3000 rpm) to obtain plasma, which was diluted with ELISA buffer to determine the levels of sex steroid hormones. Refer to supplementary information for specific operation methods (Appendix B).

### 2.5. Gene Expression Analysis

Total mRNA was extracted from brain, liver and gonad tissues by the TRIzol reagent protocol (Tiangen Biotech, Beijing, China). The first-strand cDNA was synthesized by the FasQuant RTase kit (Tiangen Biotechnology, Beijing, China), gene expression was measured by the SYBR Green PCR Master Mix kit, and β-actin was used as the housekeeping gene. See Supplementary Materials information (Table S1) for details of primer sequences and RT-qPCR.

### 2.6. Statistical Analysis

SPSS 17.0 software (SPSS, Chicago, IL, USA) and GraphPad Prism 6.0 (GraphPad Software Inc., San Diego, CA, USA) were used for data analysis and image drawing. Significant differences between the control and the CyB-treated groups were analyzed by one-way ANOVA followed by Dunnett’s test; a *p* value < 0.05 indicated statistically significant differences.

## 3. Results

### 3.1. Development of Zebrafish

**Figure 1:**
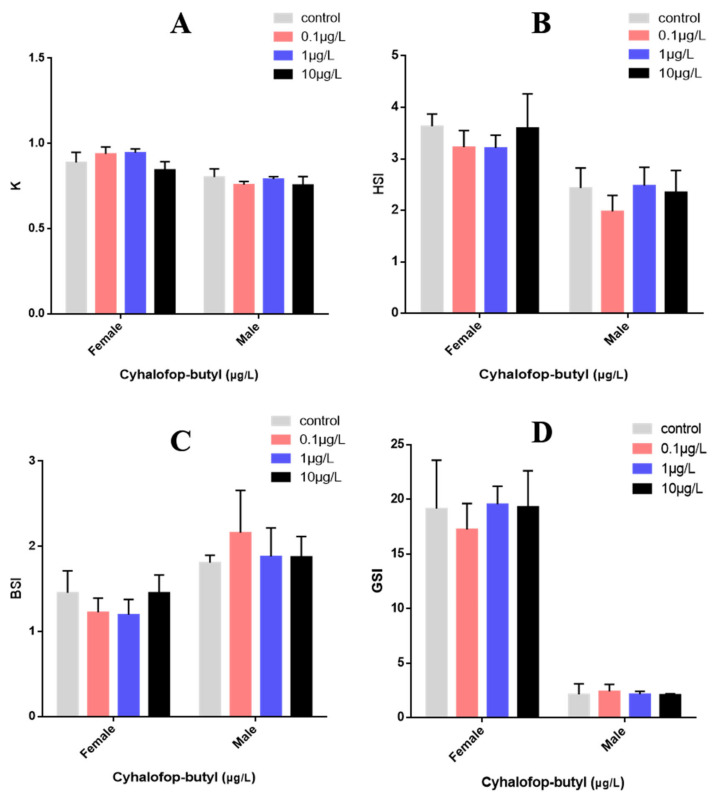
Effects of CyB on the growth and development of adult zebrafish after 150 days. (**A**) K = weight (g)/length^3^(cm) × 100. (**B**) HSI = liver weight × 100/total weight. (**C**) BSI = brain weight × 100/total weight. weight. (**D**) GSI = gonad weight × 100/total weight. No significant differences were measured between control and exposure groups (*p* < 0.05).

The effects of CyB exposure for 150 days on the growth and development of adult zebrafish are shown in Figure 1. K-factor, BSI, HSI and GSI were not significantly affected.

### 3.2. Measurement of Zebrafish Fertility

**Figure 2:**
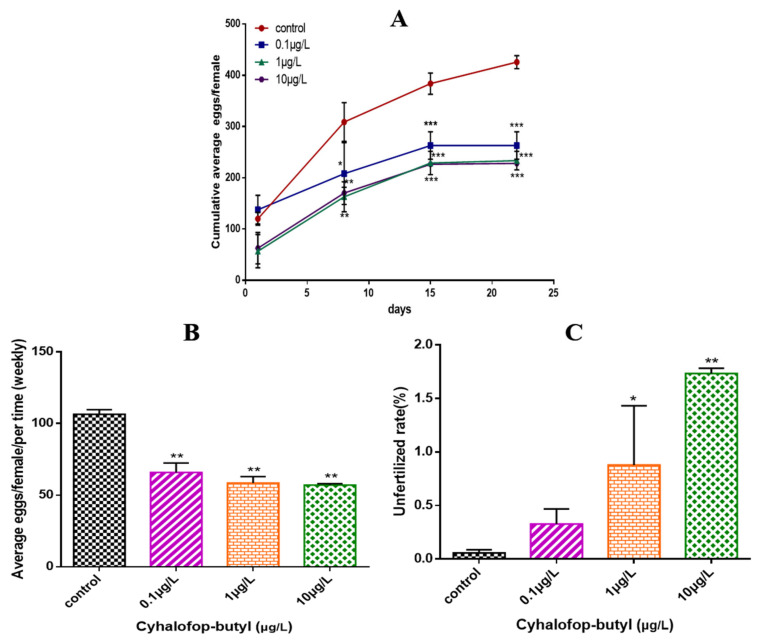
Effects of CyB on the fertility of zebrafish. (**A**) Cumulative average number of eggs per female. (**B**) Average number of spawning eggs per female and per week. (**C**) Rate of unfertilized eggs during the 150 d of exposure. The rate of unfertilized eggs was calculated as the cumulative number of unfertilized eggs × 100/total spawning eggs. Values are shown as the mean ± SD of three replicates per treatment (* *p* < 0.05; ** *p* < 0.01; *** *p* < 0.001).

After exposure to CyB, the fecundity of zebrafish decreased significantly in a concentration-dependent manner (Figure 2A), and the number of unfertilized eggs exposed to 1 and 10 µg/L CyB also increased (Figure 2B).

### 3.3. Gonadal Histological Examination

**Figure 3:**
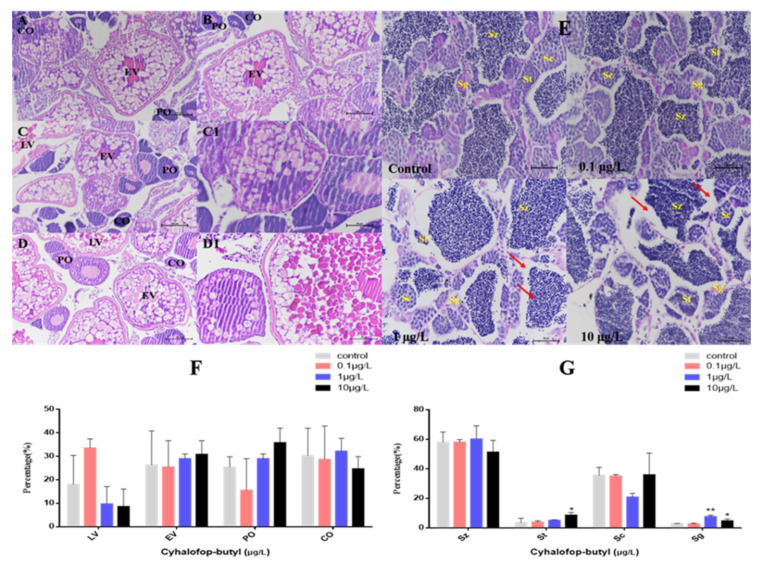
Histological observation of the gonads of zebrafish exposed to CyB for 150 d. Females: (**A**) control; (**B**) 0.1 µg/L; (**C**,**C1**) 1 µg/L; (**D**,**D1**) 10 µg/L. The oocytes in the ovaries included perinucleolar oocytes (PO), cortical alveolar oocytes (CO), early vitellogenic oocytes (EV) and late vitellogenic oocytes (LV). ((**A**–**D**) 200× magnification; (**C1**,**D1**) 400× magnification). Males (**E**) control; 0.1 µg/L; 1 µg/L; 10 µg/L. The spermatocytes included spermatogonia (Sg), spermatocytes (Sc), spermatids (St) and spermatozoa (Sz), Red arrows indicate a widened interstitial space (400× magnification). Percentage (%) of different stages of oocytes in females (**F**) and spermatogenic cells in males (**G**). The results are presented as the mean ± SD of three replicates (* *p* < 0.05; ** *p* < 0.01).

In female ovaries, there were no significant changes in perinuclear oocytes (PO), cortical alveolar oocytes (CO), early vitellogenic oocytes (EV) and late vitellogenic oocytes (LV) all groups. In male testis, CyB treatment decreased the percentage of sperm (mature sperm cells) and spermatocytes, but the change was not significant (Figure 3F). The percentage of sperm cells (immature sperm cells) induced by 10 μg/L CyB increased significantly. A significant increase in the relative percentage of spermatogonia was observed in the 1 and 10 μg/L CyB groups (Figure 3G). The female gonads were mainly characterized by the separation of the follicular wall and yolk or the detachment of the outer membrane (Figure 3A–D), while the male gonads were primarily characterized by widened stroma and decreased sperm number (Figure 3E).

### 3.4. Contents of Sex Steroid Hormones and VTG

**Figure 4:**
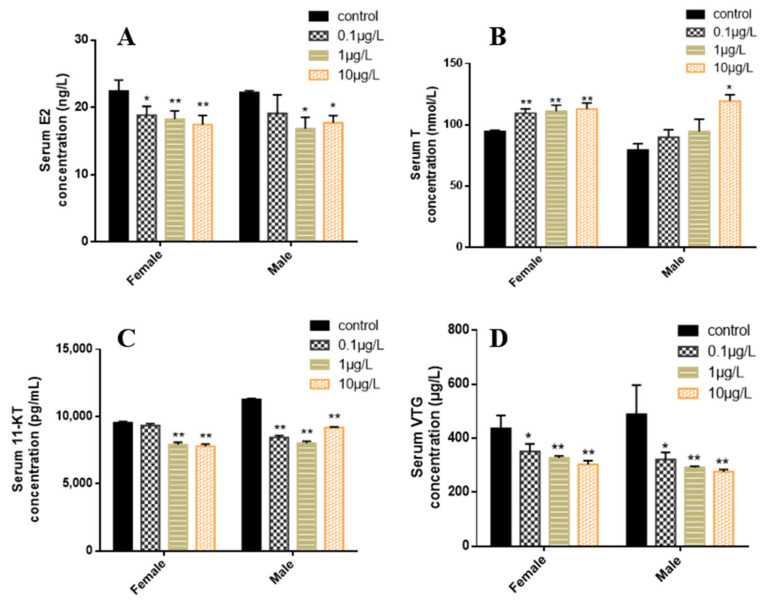
Effects of CyB exposure for 150 d on sex steroid hormones and VTG in F0 zebrafish. (**A**) 17β-estradiol (E2), (**B**) testosterone (T), (**C**) 11-ketotestosterone (11-KT) and (**D**) vitellogenin. The results are presented as the mean ± SD of three replicates (* *p* < 0.05; ** *p* < 0.01).

In female zebrafish plasma, the E2 level decreased significantly in all treatment groups, while in males, it significantly decreased only in the 1 and 10 μg/L concentration groups (Figure 4A). The T level in female zebrafish significantly increased in all CyB-exposed groups, while in male zebrafish, it significantly increased in the highest concentration group (10 μg/L) (Figure 4B). The levels of 11-KT and VTG were significantly decreased in the 1 and 10 μg/L concentration groups (Figure 4C,D).

### 3.5. Gene Expression Alteration Related to the HPGL Axis

**Figure 5:**
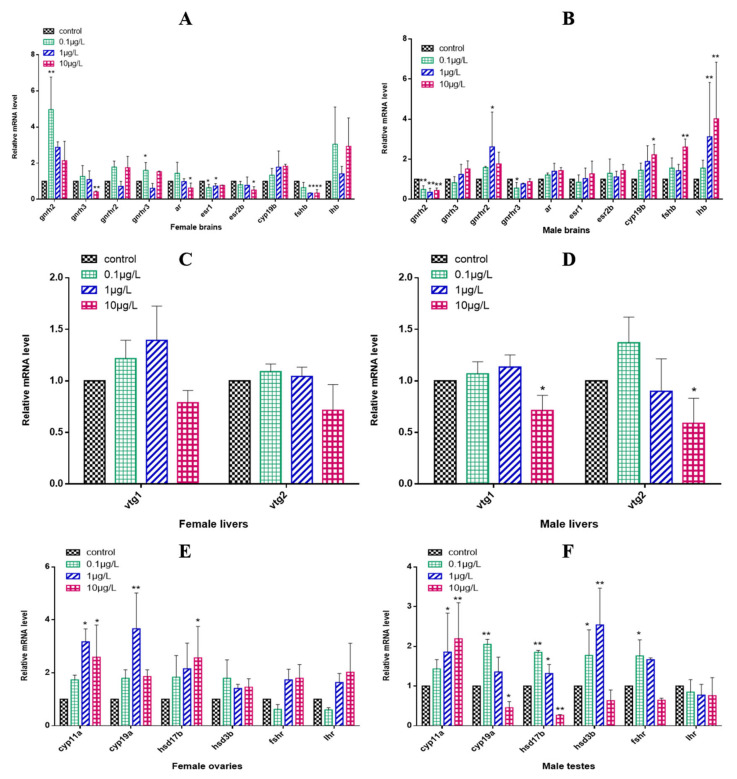
Transcription of HPGL axis-related genes in F0 zebrafish after 150 days of exposure to CyB. Females: (**A**) brain, (**C**) liver, (**E**) gonads; Males: (**B**) brain, (**D**) liver, (**F**) gonads. The results are presented as the mean ± SD of three replicates (* *p* < 0.05; ** *p* < 0.01).

In the female zebrafish brains, the expression of *gnrhr2* and *lhb* showed no significant changes after 0.1, 1 or 10 μg/L CyB exposure for 150 d compared with the control group, while the expressions of *gnrh2* and *gnrhr3* significantly increased in the 0.1 μg/L treatment group; *gnrh3*, *ar* and *esr2b* were significantly downregulated in the highest concentration treatment group (10 μg/L) (Figure 5A). In the male brains, the levels of *gnrh3*, *ar*, *esr1* and *esr2b* were increased but not significantly changed compared to the controls, even in the highest concentration group, while *gnrh2* expression was significantly downregulated in all treatment groups, and *lhb* level was significantly increased in the 1 and 10 μg/L groups. The expression of *cyp19b* and *fshb* was significantly upregulated in the 10 μg/L CyB group (Figure 5B). Neither *vtg1* nor *vtg2* levels were significantly changed in the livers of females in the CyB-treated group compared to the control group (Figure 5C). In male livers, 10 μg/L of CyB significantly downregulated the expression levels of *vtg1* and *vtg2* (Figure 5D). In female gonads, compared with the control group, the expression of *hsd3b*, *fshr* and *lhr* did not change significantly, whereas the expression of *cyp11a* was significantly upregulated in the 1 and 10 μg/L CyB groups, and that of *cyp19a* and *hsd17b* was significantly upregulated in the highest concentration treatment group (10 μg/L) (Figure 5E). In male gonads, *cyp11a* was significantly upregulated in the 1 and 10 μg/L treatment groups, *cyp19a* and *fshr* were significantly upregulated in the 0.1 μg/L CyB group, and *cyp19a* and *hsd17b* were significantly downregulated in the 10 μg/L CyB group. The expression of *hsd17b* and hsd3b was significantly upregulated in the 0.1 and 1 μg/L CyB groups (Figure 5F).

## 4. Discussion

The reproductive capacity of aquatic organisms is of great importance to the maintenance of population density and the stability of the aquatic ecosystem [35,36]. However, many environmental pollutants, especially pesticides (such as glyphosate, dimethylbenzene and pretilachlor), have been reported to have significant inhibitory effects on the reproductive capacity of fish in rivers and oceans [37,38,39]. Likewise, in this study, cyhalofop-butyl was found to have a significant inhibitory effect on the reproductive capacity of zebrafish. During the whole life cycle of zebrafish embryos exposed to CyB, the gonad index (GSI) of both male and female zebrafish showed a downward trend. The herbicide modified the levels of sex hormones and vitellogenin to varying degrees and also disturbed the relative expression of genes related to the HPGL axis, affecting the reproduction of parent zebrafish and thus leading to a decline in reproductive capacity and fertilization.

The results showed that after exposure to CyB at the environment-related concentration of 0.01 μg/L for 120 d, the average accumulated egg production of zebrafish decreased significantly. Severe reproductive inhibition was observed in the treatment groups with high concentrations of CyB (1 and 10 μg/L), leading to a decrease in the relative oviposition of zebrafish of 45% and 46% respectively, during the whole exposure period. Although GSI is usually a quantitative indicator of sexual maturity and ovarian development in vertebrates [40,41], the value of GSI is often affected by other factors, such as the oviposition cycle; consequently, the determination of GSI may not be as sensitive as a gonad histology analysis in the evaluation of reproductive toxicity. In this study, it was observed that compared to the control group, the fecundity in the 10 μg/L CyB-exposed group decreased significantly and the percentage of LV decreased by 9%, while the GSI value did not change significantly. A similar change was observed in zebrafish after 21 days of exposure to 1 mg/L of Boscalid. Female fertility decreased, and the oocyte stage distribution in the ovary was abnormal, but the GSI value did not change [34]. At the same time, CyB exposure also significantly affected the reproductive system of male zebrafish. We found that exposure to 1 μg/L and 10 μg/L of CyB resulted in the inhibition of spermatogenesis in the testis of male fish. Inhibition of parental spermatogenesis may affect the egg fertilization rate. Similarly, our study found that chronic exposure to 200 μg/L of azoxystrobin also led to the inhibition of spermatogenesis in male zebrafish and a decrease in the fertilization rate [42]. In addition, CyB exposure also caused tissue damage in zebrafish gonads, notably, the separation in female gonads of the follicle wall from the yolk, loss of the outer membrane, enlargement of the male gonadal stroma and a decrease in the number of spermatozoa.

This study found that the E2 content in zebrafish exposed to CyB decreases significantly, while that of T increased, indicating that the steady state of sex steroid hormones was altered. In teleost, the content and balance of E2 and T sex steroid hormones are considered to play an important role in sex differentiation and reproduction [43]. In addition, VTG provides the energy and nutrients needed for the growth and development of newborn fish embryos and young fish [44]. In fish, E2 can induce the production of VTG and regulate the synthesis of VTG and related gene expression in the liver [45,46]. Therefore, the decrease in VTG content in male and female zebrafish could be induced by the significant decrease of E2, which could also affect male and female zebrafish vitellogenesis in the eggs [47]. This is consistent with the decrease in the expression of *vtg1* and *vtg2* in the liver of male and female fish. Therefore, our results show that CyB disrupts the hormonal balance and affects the reproduction of zebrafish.

The HPGL axis regulates the physiological process of fish gametogenesis, and the content of sex hormones is associated with changes in sex hormone synthesis-related genes regulated by the HPGL axis [48,49]. GnRH is a biosynthetic gonadotropin (GnHs) in the hypothalamus, regulated by the HPGL axis [50,51]. The increased expression of *gnrh2*, *gnrhr2* and *gnrhr3* in females and the upregulation of *gnrhr3* and *gnrhr2* in males are consistent with a decrease in E2 production, which indicates that CyB can directly regulate the content of GnRH, thus affecting the secretion of GnHs. In the process of regulation, the pituitary gland synthesizes and secretes key hormones for the HPGL axis, such as follicle-stimulating hormone (FSH) and luteinizing hormone (LH), which promote ovarian development and differentiation and regulate gamete formation and steroid hormone synthesis [52,53]. FSH is a glycoprotein that can promote E2 synthesis, gonadal hormone secretion and puberty spermatogenesis [54], LH stimulates the synthesis of androgens and the secretion of progesterone [34,55,56]. Therefore, the decrease of *fsh* in zebrafish brain may inhibit the synthesis of E2 in female fish, resulting in a change of LV levels in the ovary and the subsequent decrease of fecundity. The increase of *fshb* and *lhb* expression in male zebrafish may affect gonad development and change the percentages of St and Sg. In addition, the biosynthesis process of steroid hormones is directly related to steroid synthase. Cholesterol is converted into testosterone by a series of enzymes (*cyp11a*, *hsd3b*, *cyp17* and *hsd17b* encode steroid synthases) and finally into estradiol by an aromatase (encoded by *cyp19a*). Therefore, changes in the expression of genes related to steroid-producing enzymes may interfere with the balance of sex hormones [26,57]. In addition, *hsd17b* catalyzes the conversion of androstenedione to T, which is then converted to 11-KT [58]. The upregulation of *hsd17b* in female ovaries and the up- and downregulation of *hsd17b* in male testes showed that CyB interfered with the steroid pathway and damaged the biosynthesis of sex hormones, thus increasing the level of T and decreasing that of 11-KT and destroying the reproductive system of male zebrafish. Aromatase (CYP19) is a crucial enzyme that catalyzes the conversion of androgen to estrogen in fish. It regulates sex differentiation and the reproductive behavior of most teleost fish by influencing E2 synthesis. *cyp19a* is mainly expressed in the gonads, and *cyp19b* is mainly expressed in the brain [59,60]. In this study, *cyp19b* was more highly expressed in the male brain, indicating that the transformation from E2 to T was increased, resulting in a significant decrease in E2 and a significant increase in T in the plasma. Therefore, we speculate that the decrease in *cyp19a* expression in the gonads of male fish prevented testosterone from being converted into estradiol, which led to the decrease in E2 synthesis in the plasma and the increase in testosterone content. Previous studies reported similar results, showing that exposure to azoxystrobin led to a decrease in *cyp19a* expression in female zebrafish ovaries and to a decrease in estradiol content in female zebrafish [43]. Tebuconazole suppressed the expression of *cyp19a* in the HPGL axis in zebrafish and decreased the content of estradiol in female fish [61]. Since fish offspring and their parents may continue to live in the same water environment in nature [62], when parents are exposed to pollutants, the offspring may be affected not only by direct exposure but also by parental exposure [63,64,65,66,67]. Therefore, the effect of CyB on zebrafish reproduction deserves attention.

## 5. Conclusions

The results of this study showed that life cycle exposure to CyB negatively affects the reproduction of adult zebrafish. Changes in the mRNA expression of HPGL axis-related markers may be the molecular mechanism that caused reproductive damage in zebrafish. These data showed that long-term exposure of zebrafish to CyB concentrations related to those found in the environment had adverse effects on some indicators of reproductive health. Considering the potential reproductive effects of these herbicides and their extensive use, our study provides valuable information for the assessment of the environmental risk they pose.

## Data Availability

The study did not report any data.

## Supplementary Materials

The following are available online at https://www.mdpi.com/article/10.3390/toxics10090495/s1, Table S1: The sequence of primers related to the HPGL axis.

## Appendix A. Zebrafish Rearing Methods during Exposure

Zebrafish were fed 3 times a day in the following ways: 2–4 dpf, no feeding; 5–12 dpf, commercial larval feed (GEMMA Micro 75, Skretting, Tooele, UT, USA), 0.05 g/tank; 12–20 dpf, commercial Larvae feed (0.05 g/tank) and brine shrimp (0.1 g/tank); 20–150 dpf, brine shrimp, the feeding amount increases with growth (the feeding amount is based on the edible amount in 10–15 min).

## Appendix B. The Method of Sex Steroid Hormone and VTG Concentrations Measurement

The serum samples used to measure E2, T, 11-KT and VTG were centrifuged (4 ℃, 3000 rpm, 10 min), and 10 μL of the supernatant was pipetted into a 96-well microplate. Enzyme-linked immunosorbent assay (ELISA) kit (Yanjin Biological Co., Ltd., Shanghai, China) was used to dilute the supernatant twice for the following measurement. The procedure is as follows:

(1) Add 10 μL of diluted serum supernatant and 40 μL of sample diluent into antibody-coated microtiter plates.
(2) After a series of incubation, washing plates, enzyme catalysis, incubation, washing plates, color rendering and termination, the absorbance was measured at 450 nm using a microplate reader.
(3) Calculate the actual concentrations of E2, T, 11-KT and VTG of serum sample according to the standard curve.
