# Supplementary material for: Life Cycle Exposure to Cyhalofop-Butyl Induced Reproductive Toxicity in Zebrafish
Source: Toxics. 2022 Aug 25;10(9):495. doi: 10.3390/toxics10090495 (PMC9503539; doi:10.3390/toxics10090495)
Supplement: Supplementary file 1 [file toxics-10-00495-s001.zip › toxics-1884439-supplementary.pdf]

Supporting Information

Life Cycle Exposure to Cyhalofop-Butyl induces Reproductive Toxicity in Zebrafish

Manman Duan [1], Xuanjun Guo [1], Xiangguang Chen [1], Mengyu Guo [1], Hao Xu [1], Lubo Hao [1], Chengju Wang [1,*] and Yang Yang [2,*]

[1] Innovation Center of Pesticide Research, Department of Applied Chemistry, College of Sciences,
China Agricultural University, Beijing 100193, China
[2] State Key Laboratory for Biology of Plant Disease and Insect Pests, Institute of Plant Protection,
Chinese Academy of Agricultural Sciences, Beijing 100193, China

* Correspondence: wangchengju@cau.edu.cn (C.W.); yangyang02@caas.cn (Y.Y.)

Table S1. The sequence of primers related to the HPGL axis.

Primer	sequence (5' to 3')
β-actin F	TGGACTCTGGTGATGGTGTGAC
β-actinR	GAGGAAGAAGAGGCAGCGGTTC
gnrh2 F	GGTCTCACGGCTGGTATCCT
gnrh2 R	TGCCTCGCAGAGCTTCACT
gnrh3 F	TGGTCCAGTTGTTGCTGTTAGTT
gnrh3 R	CCTGAATGTTGCCTCCATTTC
gnrhr2 F	ACAGCGTGAGCAAAACATTG
gnrhr2 R	TGAGCACAAACTCAGCATCC
gnrhr3 F	AACAGACATGATCCCGAAGG
gnrhr3 R	AGGTTCCCGAACACAAACAG
esr1 F	CCCACAGGACAAGAGGAAGA
esr1 R	CCTGGTCATGCAGAGACAGA
esr2b F	CAACAGGGAGGAAGGGAA
esr2b R	TTAGCAGATGAGCGAGCC
ar F	ACATTCTGGAGGCCATTGAG
ar R	ACGTGCAAGTTACGGAAACC
vtg1 F	CTGCGTGAAGTTGTCATGCT
vtg1 R	GACCAGCATTGCCCATAACT
vtg2 F	TACTTTGGGCACTGATGCAA
vtg2 R	AGACTTCGTGAAGCCCAAGA
cyp11a F	AATGGGAAGTATCCTGGTG
cyp11a R	CTGTAGGTCTGGCTGTCG
cyp19a F	GCTGACGGATGCTCAAGGA
cyp19a R	AAACGTCCACCACGATGCA
cyp19b F	CAGTCGTTACTTCCAGCCATTC
cyp19b R	CCGCTGTTTCTCCGTTGC
hsd17b F	ACATTCACGGCTGAGGAGTTT
hsd17b R	ATGCTGCCATACGTTTGGTC
hsd3b F	GCAACTCTGGTTTTCCACACTG
hsd3b R	CAGCAGGAGCCGTGTAGCTT
fshr F	CGTCTCTTTTGTGCACTGGA
fshr R	GTGGCAATTCCACACTTCCT
lhr F	CCTGGTCGTCCTGCTGGTT
lhr R	AAGGCTAGATGGCACATTAGAAATC
fshb F	GCAGGACTATGCTGGACAATG
fshb R	CCACGGGGTACACGAAGACT
lhb F	GGCTGGAAATGGTGTCTTCTT
lhb R	GGAAAACGGGCTCTTGTAAAC
